# Upper Urinary Tract Culture Positivity in Obstructive Ureterolithiasis—Clinical Severity, Inflammatory Biomarkers, and Early Risk Assessment

**DOI:** 10.3390/clinpract16100185

**Published:** 2026-10-08

**Authors:** Ana-Maria Ivănuță, Marius Ivănuță, Dragoș Puia, Nicolae Stoican, Diana-Carmen Cimpoeșu, Alexandra Haută, Cătălin Pricop

**Affiliations:** 1 Grigore T. Popa University of Medicine and Pharmacy Iasi, 700115 Iasi, Romania; anamariaion92@yahoo.com (A.-M.I.); dragos-puia@umfiasi.ro (D.P.); dcimpoiesu@yahoo.com (D.-C.C.); alexandra.hauta@umfiasi.ro (A.H.); catalin.pricop@umfiasi.ro (C.P.); 2 Emergency Care Department, “Sf. Spiridon” County University Emergency Hospital, 700111 Iasi, Romania; 3 Department of Urology, “Dr. C.I. Parhon” Clinical Hospital, 700503 Iasi, Romania; 4 Department of Urology, “Sf. Ioan cel Nou” County Emergency Hospital, 720033 Suceava, Romania; nicustoican@yahoo.com

**Keywords:** obstructive ureterolithiasis, procalcitonin, NEWS2, urinary tract infection, upper urinary tract culture, urinary decompression, urosepsis, risk stratification

## Abstract

**Background/Objectives:** Obstructive ureterolithiasis complicated by infection may progress rapidly to systemic deterioration, while microbiological confirmation is generally unavailable during initial assessment. This study evaluated the association of admission procalcitonin (PCT), National Early Warning Score 2 (NEWS2), and routine urinary parameters with positive upper urinary tract culture in patients requiring urgent urinary decompression. **Methods**: This retrospective single-center observational study included 240 adults admitted with obstructive ureterolithiasis who underwent upper urinary tract decompression by ureteral stenting or percutaneous nephrostomy. Demographic, clinical, laboratory, and urinary parameters obtained at admission were compared according to upper urinary tract culture status. Factors associated with culture positivity were assessed using univariable and multivariable logistic regression. Four clinically defined models were compared: a reference model containing WBC, CRP, urinary nitrites, and proteinuria; models separately adding PCT or NEWS2; and a complete model containing both. Discrimination was compared using AUCs and the DeLong test, while model fit was assessed using likelihood-ratio tests. Internal validation was performed using 1000 bootstrap resamples. **Results**: Positive upper urinary tract cultures were identified in 86 patients (35.8%). In the complete model, PCT per doubling (aOR 1.820; 95% CI 1.502–2.205; *p* < 0.001) and NEWS2 (aOR 1.167; 95% CI 1.014–1.344; *p* = 0.031) remained independently associated with culture positivity. The reference model had an AUC of 0.691. Adding PCT increased the AUC to 0.831 (ΔAUC 0.140; *p* < 0.001), whereas adding NEWS2 alone resulted in an AUC of 0.720 (ΔAUC 0.029; *p* = 0.085). The complete model achieved an apparent AUC of 0.839 and an optimism-corrected AUC of 0.821. After PCT was included, NEWS2 improved overall model fit (*p* = 0.029) and produced an additional increase in AUC (ΔAUC 0.007; *p* = 0.448). **Conclusions**: Admission PCT and NEWS2 were independently associated with positive upper urinary tract culture The combined model showed good discrimination, although external validation is required before clinical application. Urinary nitrite positivity retained an independent association only in the exploratory cut-off-based analysis.

## 1. Introduction

Obstructive ureterolithiasis represents a complex clinical condition in which mechanical obstruction and infection may coexist, leading to rapid clinical deterioration and risk of severe complications, including urosepsis and organ dysfunction. Stone disease itself is increasingly recognized as a multifactorial process involving not only physicochemical factors but also inflammatory pathways, epithelial injury, and recurrent infectious episodes, which may contribute to renal damage over time [1,2].

When urinary obstruction is accompanied by infection, timely decompression is essential to prevent progression toward systemic inflammatory response and septic complications. However, the optimal timing of intervention remains poorly defined, and clinical decision-making often relies on nonspecific findings and heterogeneous presentations [1,3]. This variability reflects the complex interplay between pathogen-related factors and the host inflammatory response, which ultimately determines clinical severity rather than infection alone [4].

In clinical practice, microbiological confirmation through urine or blood cultures remains the reference standard for diagnosing infection, yet results are typically delayed and may not reflect the early severity of the disease. Conventional inflammatory markers such as C-reactive protein and leukocyte count are widely used but lack specificity, as they may be elevated in a variety of noninfectious conditions [4,5]. This limitation underscores the need for more reliable early biomarkers capable of distinguishing clinically relevant bacterial infection.

Procalcitonin (PCT) has emerged as a promising biomarker in this context, given its association with bacterial infection and systemic inflammatory response. Elevated PCT levels have been correlated with severe urinary tract infections, urinary obstruction, and the need for urgent intervention, suggesting a potential role in early clinical decision-making [4,6]. Moreover, higher PCT levels have been observed in patients with complicated infections and urosepsis, supporting its use as an indicator of infection severity. Nevertheless, the prognostic value of PCT remains variable across studies, and its performance may be influenced by patient characteristics, comorbidities, and infection profiles [7,8,9].

In parallel, clinical scoring systems such as the National Early Warning Score 2 (NEWS2) provide a standardized assessment of physiological deterioration and have been widely implemented to detect clinical instability early. However, their role in patients with obstructive ureterolithiasis complicated by infection remains unclear, particularly regarding microbiological confirmation and infection-specific outcomes [10].

Clinical management of obstructive ureterolithiasis remains challenging because no admission-based model currently integrates clinical severity assessment with infection-related biomarkers for early risk stratification. Although parameters such as procalcitonin and physiological scoring systems may individually provide useful diagnostic and prognostic information, data on their combined value in predicting culture-positive infection and severe in-hospital outcomes remain limited [11,12,13].

The early assessment of patients with obstructive ureterolithiasis is often challenging because clinical presentation may vary considerably, while microbiological confirmation is rarely available when initial management decisions are made. Admission evaluation therefore relies on a combination of clinical status, inflammatory biomarkers, and urinary findings. PCT may provide information regarding the systemic response to bacterial infection, whereas NEWS2 offers a standardized assessment of acute physiological deterioration. Urinary nitrites, WBC, and CRP remain readily available parameters, although none provides sufficient information when interpreted in isolation.

Evidence regarding the combined interpretation of these parameters in patients requiring urgent upper urinary tract decompression remains limited. The primary objective of the present study was to evaluate the associations of admission PCT, NEWS2, and routine urinary and inflammatory findings with positive culture obtained directly from the obstructed collecting system. We also examined the performance of models incorporating PCT and NEWS2, separately and together, to better define their contribution to admission-based risk assessment. In-hospital outcomes were recorded as secondary descriptive variables and compared according to upper urinary tract culture status.

## 2. Materials and Methods

### 2.1. Study Design and Patient Selection

This retrospective observational study was conducted in a single-center university-affiliated hospital and included adult patients admitted between January 2023 and January 2024 with obstructive ureteral lithiasis requiring upper urinary tract decompression. Urinary drainage was achieved either by retrograde ureteral stent insertion (double-J stent) or percutaneous nephrostomy, according to the attending urologist’s clinical judgment and anatomical considerations.

Obstruction was confirmed by imaging studies, including ultrasonography and/or computed tomography. Clinical, demographic, laboratory, and urinary parameters were extracted from electronic medical records and analyzed in relation to positive upper urinary tract culture.

Patients undergoing elective double-J stent placement as a preparatory step for subsequent ureteroscopic treatment were excluded, as these procedures were not performed in an acute obstructive setting. Patients requiring upper urinary tract decompression for non-lithiasic causes of obstruction were also excluded. Additional exclusion criteria included insufficient documentation to establish the precise indication for ureteral stent placement or percutaneous nephrostomy, incomplete clinical or laboratory data required for the planned analyses, and any systemic antibiotic exposure before collecting the upper urinary tract urine sample. The latter criterion was applied to minimize the risk of reduced microbiological yield and false-negative culture results.

During the study period, no major changes were introduced in the institutional approach to urgent urinary decompression, upper urinary tract urine sampling, microbiological processing, or the timing of empirical antibiotic administration relative to sample collection.

### 2.2. Data Collection and Study Variables

Clinical outcomes, including ICU admission, vasopressor support, in-hospital mortality, and length of hospital stay, were recorded as secondary descriptive variables and compared according to upper urinary tract culture status. Urine samples were obtained directly from the obstructed collecting system at the time of urinary decompression, either during retrograde ureteral stent placement or through percutaneous nephrostomy. Bladder urine cultures, when available, were recorded separately and were not used to define the primary endpoint.

Upper urinary tract samples were quantitatively inoculated using a calibrated 10 μL loop onto Columbia sheep blood agar and MacConkey agar and incubated aerobically overnight at 37 °C. Bacterial growth was quantified as CFU/mL. Identification was based on colony morphology and Gram staining, followed, as appropriate, by conventional biochemical testing, the MicroScan WalkAway system (Beckman Coulter, Brea, CA, USA), the BD Phoenix system (Becton, Dickinson and Company, Sparks, MD, USA), or MALDI-TOF mass spectrometry. Polymicrobial culture was defined as the isolation of two recognized uropathogens at significant counts. Mixed growth without a predominant clinically relevant organism was considered contamination and excluded from the analysis. Antimicrobial susceptibility testing was performed by disk diffusion on Mueller–Hinton agar or automated broth microdilution and interpreted according to EUCAST criteria. Polymicrobial culture was defined as the isolation of two recognized uropathogens, each at ≥10^5^ CFU/mL. Growth of three or more organisms without a predominant pathogen was considered contamination, and these samples were excluded from the analysis.

Bacterial identification was performed according to the routine laboratory protocol. Antimicrobial susceptibility testing was automated and based on minimum inhibitory concentration determination, with interpretation according to the EUCAST clinical breakpoints applicable at the time of testing.

Laboratory parameters collected at admission included leukocyte count, CRP, PCT, urea, and eGFR. Urinary abnormalities, including nitrituria and proteinuria, were also evaluated. Clinical severity at presentation was assessed using NEWS2. In the context of this study, NEWS2 was interpreted as an admission-based measure of acute physiological compromise, intended to complement inflammatory biomarkers and urinary findings in the early identification of patients with culture-positive infected obstruction, rather than as a standalone diagnostic tool for urinary infection [14].

Continuous variables were analyzed based on their distributional characteristics and are presented as mean ± standard deviation or median, as appropriate. Categorical variables are expressed as absolute frequencies and percentages.

### 2.3. Statistical Analysis

To identify factors associated with positive upper urinary tract culture, univariable logistic regression analyses were initially performed. The six candidate variables—log_2_-transformed PCT, NEWS2, urinary nitrite positivity, proteinuria, WBC, and CRP—were selected based on their clinical relevance, biological plausibility, and availability during the initial assessment. All six variables were entered simultaneously and retained in the complete model, irrespective of their adjusted statistical significance. No automated or stepwise variable-selection procedure was applied.

Regression coefficients were reported as ORs with 95% confidence intervals (95% CIs). Statistical significance was defined as a two-sided *p*-value < 0.05.

Because PCT values showed a markedly right-skewed distribution, the linearity of its relationship with the log-odds of positive upper urinary tract culture was assessed using the Box–Tidwell procedure. PCT was log2-transformed before inclusion in the multivariable model. The corresponding odds ratio was interpreted per doubling of PCT.

To quantify the incremental contribution of PCT and NEWS2 beyond routinely available inflammatory and urinary findings, an additional analysis based on four clinically defined logistic regression models was performed. Model 1 served as the reference model and included WBC, CRP, urinary nitrite positivity, and proteinuria. Model 2 added log_2_-transformed PCT to the reference model, whereas Model 3 added NEWS2. Model 4 represented the complete model and included the four reference variables together with log_2_-transformed PCT and NEWS2. The same 240 complete cases were used in all four models.

### 2.4. Model Performance Assessment

The discriminative performance of the multivariable model was assessed using ROC curve analysis. Model discrimination was quantified by calculating the area under the ROC curve (AUC) and the corresponding 95% confidence interval.

Model calibration was evaluated using the Hosmer–Lemeshow goodness-of-fit test. Calibration plots comparing predicted and observed probabilities were additionally generated to visually assess the agreement between predicted risk estimates and observed outcomes across the probability spectrum. Differences between correlated AUCs were evaluated using the DeLong method. Nested models were compared using likelihood-ratio tests.

Internal validation was performed using bootstrap resampling with 1000 repetitions. In each bootstrap sample, the model was refitted, and its discrimination was assessed in both the bootstrap sample and the original cohort. The mean difference between these estimates was used to quantify optimism and calculate an optimism-corrected AUC.

Overall model fit was assessed using the Omnibus Tests of Model Coefficients.

To improve clinical interpretability, a secondary categorical analysis was also performed. NEWS2 was categorized using the clinically relevant threshold of ≥5. An exploratory PCT cut-off was identified by receiver operating characteristic analysis using the Youden index. The resulting categorical variables were entered into a multivariable logistic regression model together with the remaining candidate predictors. This analysis was considered exploratory and did not replace the primary continuous-variable analysis.

All statistical analyses were performed using IBM SPSS, version 26.0 (IBM Corp., Armonk, NY, USA). All tests were two-tailed, and statistical significance was considered at *p* < 0.05 throughout the analysis.

## 3. Results

**Figure 1:**
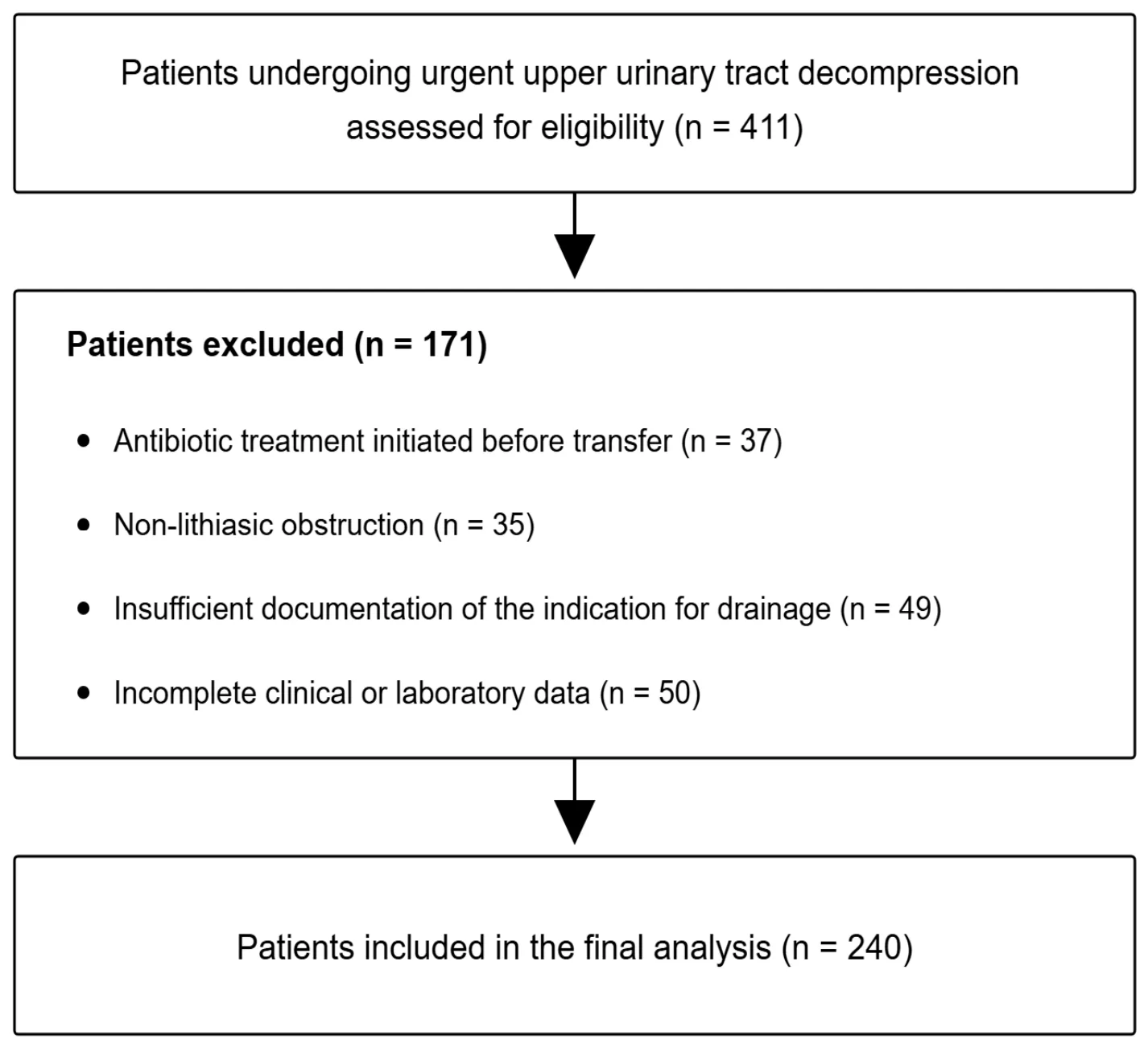
Flow diagram of patient selection.

During the study period, 411 patients undergoing urgent upper urinary tract decompression were assessed for eligibility. A total of 171 patients were excluded according to the predefined criteria, resulting in a final cohort of 240 patients as can be observed in Figure 1.

The study population demonstrated a substantial burden of cardiovascular and metabolic comorbidities, alongside a marked inflammatory response at admission. More than one-quarter of patients presented with a NEWS2 score ≥5, suggesting clinically significant systemic involvement at initial evaluation.

Positive upper urinary tract cultures were identified in over one-third of cases. Despite urinary decompression, a relevant proportion of patients subsequently required vasopressor support and intensive care monitoring, reflecting the potential severity and systemic impact of obstructive urinary tract infections in the acute care setting. As shown in Table 1, *Escherichia coli* was the predominant microorganism, while polymicrobial cultures were uncommon and involved the concurrent isolation of *E. coli* and *Enterococcus* spp.

Baseline demographic, clinical, laboratory, microbiological, and outcome characteristics of the study population are summarized in Table 2.

As shown in Table 3, patients with positive upper urinary tract cultures had higher admission NEWS2, WBC, CRP, and PCT values than culture-negative patients.

Urinary findings suggestive of infection were also more frequently identified in the culture-positive group, with significantly higher rates of positive urinary nitrites and proteinuria.

While baseline demographic characteristics and major comorbidities were generally comparable between groups, culture-positive patients more frequently required vasopressor support and had longer hospital stays, suggesting a more severe systemic clinical course despite urinary decompression.

No statistically significant differences were observed in age, sex, renal function parameters, history of urinary tract infection, or intensive care unit admission.

Table 4 summarizes the univariable logistic regression analysis for predictors of positive upper urinary tract cultures. Higher levels of inflammatory biomarkers, including leukocyte count, CRP, and procalcitonin, were associated with increased odds of positive upper urinary tract culture. Increased NEWS2 scores, positive urinary nitrites, and proteinuria were also significantly associated with positive cultures.

In contrast, demographic characteristics, medical comorbidities, and baseline renal function parameters were not significantly associated with positive upper urinary tract culture in the univariable analysis.

Table 5 presents the multivariable logistic regression model for positive upper urinary tract culture. Because the linearity-in-the-logit assumption was not met for untransformed PCT values, PCT was log2-transformed before inclusion in the model. The corresponding odds ratio therefore represents the change in the odds of positive upper urinary tract culture for each doubling of PCT. After adjustment, log_2_-transformed PCT and NEWS2 remained independently associated with positive upper urinary tract culture. Urinary nitrite positivity, proteinuria, WBC, and CRP did not retain statistical significance in the primary continuous-variable model.

The overall six-variable model was statistically significant (likelihood-ratio χ^2^(6) = 88.253, *p* < 0.001), with Cox–Snell and Nagelkerke R^2^ values of 0.308 and 0.422, respectively. The Hosmer–Lemeshow test did not indicate lack of fit (χ^2^(8) = 4.755, *p* = 0.783).

As shown in Table 6, an exploratory PCT threshold of 3.0 ng/mL yielded a sensitivity of 84.9% and a specificity of 69.5% for positive upper urinary tract culture. After adjustment, PCT ≥ 3.0 ng/mL was associated with higher odds of culture positivity (adjusted OR 10.881; 95% CI 5.215–22.702; *p* < 0.001). NEWS2 ≥ 5 was also independently associated with culture positivity (aOR 2.802; 95% CI 1.419–5.536; *p* = 0.003). The categorical model achieved an AUC of 0.846.

**Figure 2:**
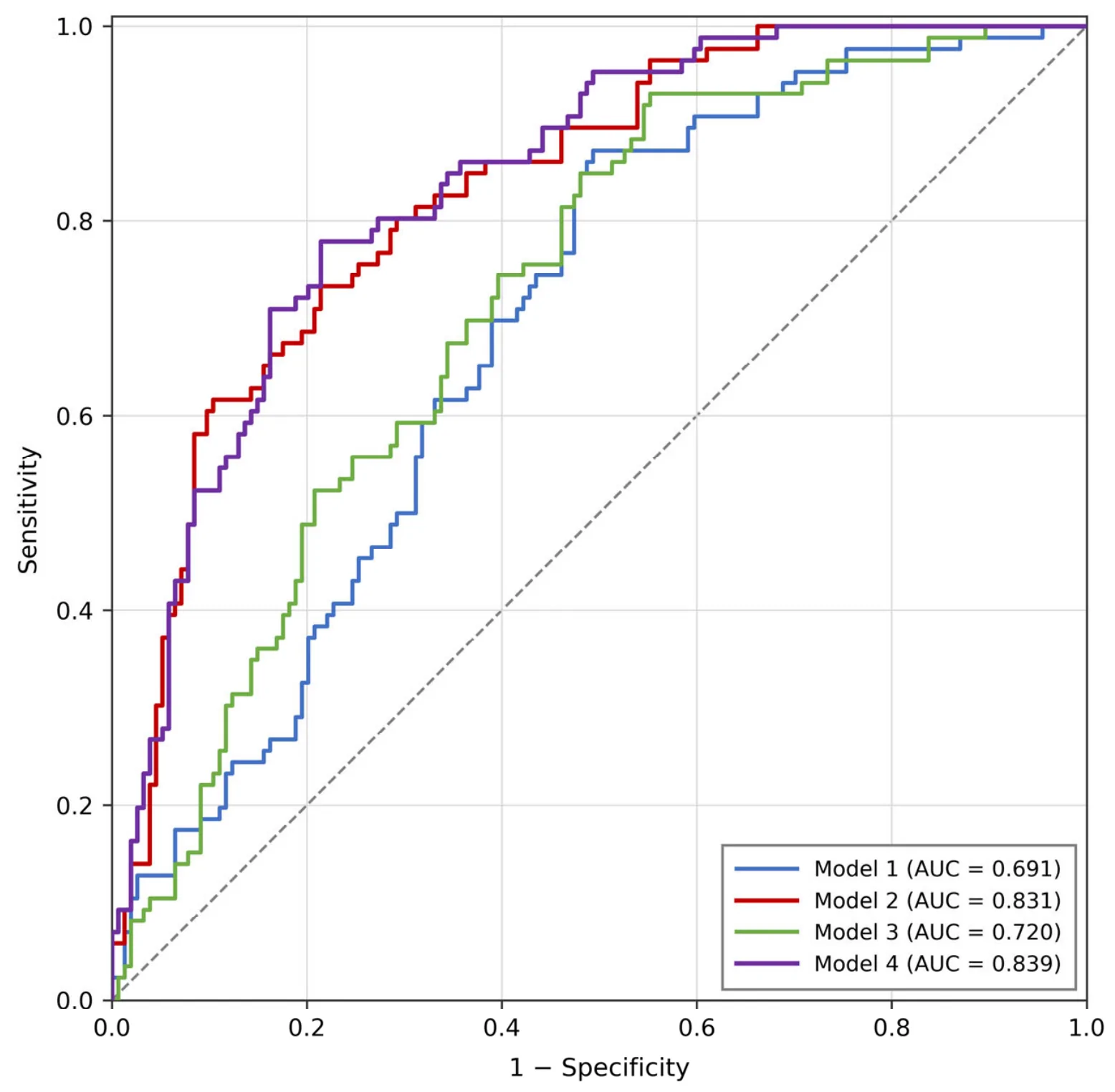
Comparative ROC curves of models incorporating routine clinical markers, PCT, and NEWS2 for predicting positive upper urinary tract culture.

Figure 2 shows the ROC curve of the final six-variable multivariable model for positive upper urinary tract culture. The model showed good apparent discrimination, with an AUC of 0.839. Bootstrap internal validation yielded a corrected AUC of 0.821, as shown in Table 6.

The incremental model comparisons are presented in Table 7. The reference model containing WBC, CRP, urinary nitrites, and proteinuria had an AUC of 0.691 (95% CI 0.624–0.758). Adding log_2_-transformed PCT increased the AUC to 0.831 (95% CI 0.780–0.883), corresponding to an absolute increase of 0.140 (DeLong *p* < 0.001). This addition also significantly improved overall model fit (likelihood-ratio χ^2^ = 57.321, *p* < 0.001).

Adding NEWS2 alone to the reference model produced a smaller increase in AUC, from 0.691 to 0.720 (ΔAUC 0.029; DeLong *p* = 0.085), although the likelihood-ratio test indicated improved model fit (χ^2^ = 5.631, *p* = 0.018). The complete six-variable model achieved an apparent AUC of 0.839 (95% CI 0.788–0.889) and an optimism-corrected AUC of 0.821.

When NEWS2 was added to the model already containing PCT, overall model fit improved (likelihood-ratio χ^2^ = 4.759, *p* = 0.029), but the additional increase in AUC was limited and not statistically significant (ΔAUC 0.007; DeLong *p* = 0.448). Conversely, adding PCT to the model containing NEWS2 increased the AUC by 0.119 (DeLong *p* < 0.001). The complete model retained an optimal predicted-probability cut-off of 0.372, with a sensitivity of 77.9% and a specificity of 78.6%.

**Figure 3:**
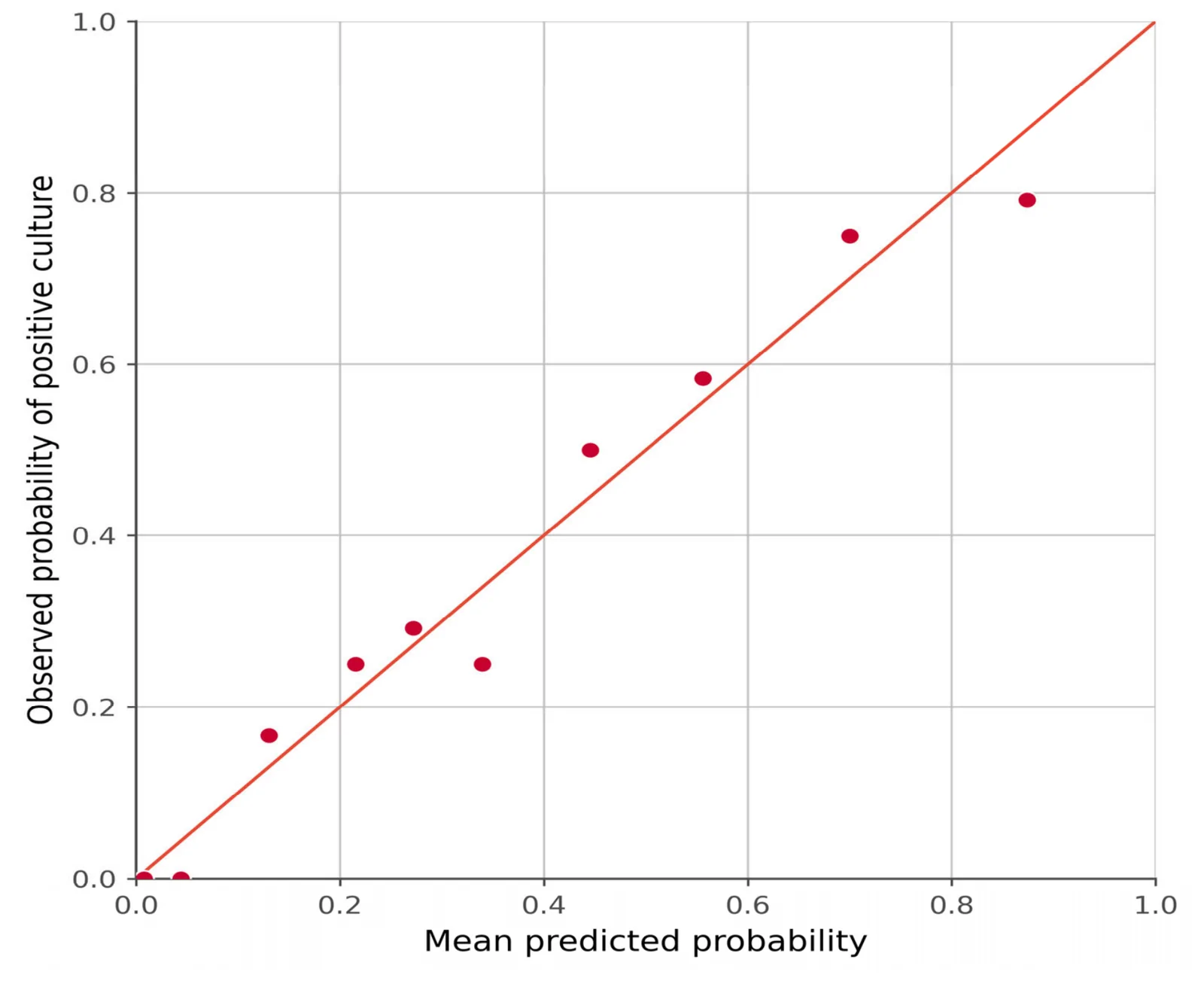
Calibration plot of the multivariable prediction model.

The final six-variable multivariable mode included log_2_-transformed procalcitonin, NEWS2 score, urinary nitrite positivity, proteinuria, white blood cell count, and C-reactive protein. Predicted probabilities from the model were used to construct the ROC curve. The optimal cut-off was selected using the Youden index. AUC = area under the curve; CI = confidence interval; PCT = procalcitonin; NEWS2 = National Early Warning Score. Internal validation was performed using 1000 bootstrap resamples. Figure 3 presents the calibration plot of the multivariable prediction model. Overall, the predicted probabilities showed reasonable agreement with the observed probability of positive upper urinary tract culture across risk deciles. The model also demonstrated acceptable calibration according to the Hosmer–Lemeshow goodness-of-fit test.

## 4. Discussion

In this retrospective cohort of patients with obstructive ureterolithiasis requiring upper urinary tract decompression, culture-positive cases exhibited a distinct clinical and biological profile at admission. Compared with culture-negative patients, they had higher NEWS2 scores, leukocyte counts, CRP and procalcitonin concentrations, and a higher prevalence of urinary nitrite positivity and proteinuria. Positive upper urinary tract culture was also associated with a higher requirement for vasopressor support and prolonged hospitalization, indicating a closer relationship with systemic disease burden and subsequent clinical complexity.

In the primary continuous-variable model, log_2_-transformed PCT and NEWS2 remained independently associated with positive upper urinary tract culture, whereas urinary nitrite positivity and proteinuria did not retain statistical significance. In the exploratory cut-off-based model, PCT ≥ 3.0 ng/mL, NEWS2 ≥ 5, and urinary nitrite positivity were independently associated with culture positivity. The findings from the categorical model should be interpreted cautiously because this analysis was secondary and exploratory.

The between-group differences observed in the present study also indicate that upper urinary tract culture positivity was not merely a microbiological endpoint. Rather, it identified patients with a more pronounced inflammatory response, greater physiological compromise, and increased in-hospital treatment requirements. This pattern is consistent with the concept that, in obstructive ureterolithiasis, microbiologically confirmed infection is closely linked to the systemic consequences of the obstructive infectious process. Upper urinary tract cultures were positive in 35.8% of patients. Although higher positivity rates have been reported in cohorts specifically selected for obstructive pyelonephritis, our study included all eligible patients with obstructive ureterolithiasis requiring urgent decompression, regardless of subsequent microbiological confirmation. Consequently, the inclusion of patients with sterile obstruction or without detectable bacterial growth may partly explain the lower culture-positive rate observed in our cohort.

The incremental comparisons further support the complementary roles of PCT and NEWS2 in this clinical setting. PCT markedly improved discrimination beyond routine inflammatory and urinary findings, consistent with its closer relationship with the systemic response to bacterial infection. NEWS2 remained independently associated with culture positivity and significantly improved overall model fit when added to the PCT-containing model, indicating that it captures relevant physiological information not fully reflected by laboratory markers. Although the corresponding increase in AUC was limited, this should be interpreted in the context of a microbiological endpoint, for which NEWS2 is not a direct diagnostic measure. The complete model therefore integrates two clinically distinct dimensions: the probability of bacterial infection and the physiological response associated with infected urinary obstruction.

The categorical analysis provides a more clinically interpretable perspective for emergency assessment. Patients presenting with PCT ≥ 3.0 ng/mL had substantially higher odds of a positive upper urinary tract culture, while NEWS2 ≥ 5 identified patients with greater physiological compromise. However, the PCT threshold was derived from the present cohort and should be regarded as exploratory until validated in an independent population.

Admission procalcitonin provided the largest incremental improvement in discrimination, while NEWS2 added complementary information regarding physiological compromise. This distinction is important in obstructive ureterolithiasis, where culture results remain essential for microbiological confirmation but are rarely available when decisions regarding antibiotics, decompression, and monitoring must be made. In this setting, a combined admission profile may help identify patients who are more likely to have culture-positive infection and who may require closer post-decompression surveillance.

The contribution of NEWS2 should also be interpreted with caution. Its association with positive upper urinary tract culture probably reflects the systemic consequences of infected urinary obstruction rather than a direct ability to diagnose urinary infection. This is consistent with emergency medicine data showing that NEWS-based systems are useful for detecting clinical deterioration and sepsis-related risk. Usman et al. reported that NEWS outperformed SIRS and qSOFA in identifying severe sepsis, septic shock, and sepsis-related mortality during emergency department triage, whereas qSOFA had limited sensitivity as a screening tool [15].

The findings of Guan et al. further support this perspective. In their systematic review and meta-analysis, early warning scores were shown to be valuable for predicting clinical deterioration in emergency department and in-hospital settings, although their performance varied by clinical context, threshold, and outcome definition. In the present study, the contribution of NEWS2 likely reflects the systemic impact of infected obstruction rather than microbiological status alone [16].

The role of procalcitonin in urinary tract infection is heterogeneous and requires careful interpretation. Several studies have shown that PCT does not perform consistently as a stand-alone diagnostic test for UTI across all patient populations. In an emergency department diagnostic accuracy study, Hertz et al. found that PCT had limited overall accuracy in diagnosing UTI, although it performed better for assessing severe disease and ruling out bacteremia. This distinction is relevant to the present cohort, which included patients with obstructive ureterolithiasis requiring urgent decompression rather than patients with uncomplicated UTI [17].

Similar caution is supported by studies in older and institutionalized populations. Choi et al. reported limited diagnostic accuracy of PCT for UTI among hospitalized older adults, while Kuil et al. found that CRP and PCT were not suitable for distinguishing true UTI from asymptomatic bacteriuria in nursing home residents [11,13]. These studies argue against using PCT as an isolated marker of urinary infection. In the present study, the association between PCT and positive upper urinary tract culture should therefore be interpreted within the narrower context of obstructive urinary infection, where bacterial burden, renal involvement, and systemic inflammatory response may be more pronounced.

This interpretation is also aligned with current recommendations regarding PCT use. Available guidance emphasizes that PCT should not be used alone to diagnose sepsis but may contribute to clinical decision-making when interpreted together with clinical assessment. PCT may also reflect infection severity and may support antibiotic stewardship, particularly when serial measurements are available. In the present study, only the admission PCT was evaluated; therefore, its role should be viewed as supportive rather than definitive [9,14,18].

Previous studies evaluating inflammatory biomarkers in relation to UTI localization provide additional context. Xu et al. reported higher PCT and CRP levels in children with acute pyelonephritis than in those with lower urinary tract infection, with PCT showing better performance in identifying upper urinary tract involvement [19]. Although pediatric findings cannot be directly extrapolated to the present adult cohort, they support the biological plausibility that PCT may be more informative when infection involves the upper urinary tract.

Shi et al. also reported that PCT and CRP had moderate diagnostic performance in distinguishing upper from lower UTI among hospitalized patients. Their results are consistent with the idea that inflammatory biomarkers alone are unlikely to confer diagnostic certainty but may add value when combined with clinical and urinary variables [20]. This is consistent with the current model, in which PCT contributed alongside NEWS2 and urinary nitrite status, rather than serving as an isolated predictor.

Urinary nitrite positivity was independently associated with culture positivity only in the exploratory cut-off-based model. This association is clinically plausible, given the relationship between urinary nitrites and nitrate-reducing uropathogens, but it should be interpreted cautiously because it was not statistically significant in the primary continuous-variable model. *Enterobacterales* were identified in 67 of the 86 positive cultures (77.9%), which may explain the association between urinary nitrites and culture positivity. However, nitrites remained negative in 30 culture-positive patients. This may reflect infection with non-nitrate-reducing organisms, particularly Enterococcus spp., but also differences in bacterial load, urinary dwell time, or the sampling site. Thus, a negative nitrite test cannot reliably exclude infection in patients with obstructive ureterolithiasis. Proteinuria was associated with positive upper urinary tract culture in the univariable analysis but did not remain independently associated after adjustment, suggesting limited additional discriminatory value once systemic inflammatory markers, NEWS2, and nitrite status were considered.

The performance of the model should be interpreted with appropriate caution. The model showed good apparent discrimination, with an AUC of 0.839. Bootstrap internal validation yielded an optimism-corrected AUC of 0.821, suggesting only limited overfitting in the derivation cohort. Nevertheless, the model was developed in a retrospective single-centre population and should be considered an adjunct to early clinical assessment rather than a definitive diagnostic tool. External validation remains necessary before clinical application. Similarly, the calibration plot suggested reasonable agreement between predicted and observed probabilities, although some deviation was observed at higher predicted risk levels. Therefore, the model should be regarded as a potential adjunct to early clinical assessment rather than a definitive diagnostic instrument.

From an emergency medicine perspective, the main clinical implication is the integration of two complementary domains: infection-related biomarkers and acute physiological severity. PCT and urinary nitrites provide information on the probability of bacterial infection, whereas NEWS2 captures systemic instability. This combination may be particularly useful during initial evaluation, when the clinician must decide on antimicrobial therapy, monitoring intensity, and urgency of urological coordination before microbiological confirmation is available.

Several limitations should be acknowledged. First, the retrospective single-center design may limit the generalizability of the findings. Second, the model was not externally validated, and its performance should therefore be confirmed in independent cohorts. Third, the analysis was based on admission measurements only; consequently, dynamic changes in PCT, CRP, NEWS2, renal function, or clinical status were not assessed. Fourth, the study population was restricted to patients with obstructive ureterolithiasis requiring decompression, and the results should not be extrapolated to uncomplicated urinary tract infection, asymptomatic bacteriuria, or non-lithiasic causes of upper urinary tract obstruction. The conservative threshold of ≥10^5^ CFU/mL may have resulted in some clinically relevant infections with lower bacterial counts being classified as culture-negative. Patient selection may have influenced the composition of the cohort. Excluding patients treated before transfer may have underrepresented some previously treated or more severe referrals, while exclusions for incomplete data also included non-interpretable microbiological results, such as contaminated cultures. However, patients receiving their first empirical antibiotic dose after admission to our institution remained eligible, although treatment shortly before sampling may have reduced culture yield. Finally, blood cultures were not systematically obtained in all patients and were therefore not included in the analysis, as their incomplete and non-uniform availability could have introduced selection bias.

Despite these limitations, the study addresses a clinically relevant challenge in acute care: the early identification of patients with obstructive ureterolithiasis who are at increased likelihood of a positive upper urinary tract culture and systemic involvement. The findings support further prospective evaluation of admission-based risk stratification models that combine inflammatory biomarkers, physiological severity scores, and urinary markers. Future studies should determine whether such models can improve clinical pathways, including early antimicrobial decision-making, prioritization of decompression, and post-decompression monitoring.

## 5. Conclusions

In this cohort of patients with obstructive ureterolithiasis requiring urinary decompression, culture positivity was associated with a greater inflammatory and physiological burden at admission. PCT and NEWS2 remained independently associated with positive upper urinary tract culture in the primary analysis, while the contribution of urinary nitrites was limited to the exploratory cut-off-based analysis. The combined model showed good discrimination and acceptable calibration within the study cohort, but its clinical value remains to be established through prospective external validation.

## Figures and Tables

**Table 1:** Microbiological distribution of positive upper urinary tract cultures.

Microbiological Finding	*n* (%)
*Escherichia coli*	38 (44.2)
*Klebsiella* spp.	14 (16.3)
*Enterococcus* spp.	13 (15.1)
*Proteus* spp.	10 (11.6)
*Pseudomonas aeruginosa*	6 (7.0)
*Escherichia coli* + *Enterococcus* spp.	3 (3.5)
*Morganella* spp.	2 (2.3)

**Table 2:** Baseline characteristics of patients with obstructive ureteral lithiasis requiring urinary decompression.

Variable	Total (*n* = 240)
Demographic characteristics	
Age, years	54.59 ± 19.33
Male sex	134 (55.8%)
Body mass index, kg/m^2^	29.13 ± 6.36
Rural residence	125 (52.1%)
Medical history and comorbidities	
Previous kidney stones	126 (52.5%)
Diabetes mellitus	128 (53.3%)
Hypertension	116 (48.3%)
Chronic kidney disease	43 (17.9%)
Previous urinary tract infection	16 (6.7%)
Hospitalization within the last 30 days	29 (12.1%)
Stone and admission characteristics	
Renal pelvis	49 (20.4%)
Upper ureter	75 (31.3%)
Iliac ureter	60 (25.0%)
Pelvic ureter	56 (23.3%)
NEWS2 score	4.00 [3.00–6.00]
NEWS2 ≥ 5	66 (27.5%)
Key admission laboratory parameters	
Serum creatinine, mg/dL	1.16 [0.93–1.51]
Urea, mg/dL	40.80 [33.03–51.43]
eGFR, mL/min/1.73 m^2^	79.50 [55.00–102.75]
White blood cell count, /mm^3^	16,271.50 [13,475.25–20,914.75]
C-reactive protein, mg/L	117.50 [60.00–201.75]
Procalcitonin, ng/mL	2.87 [1.24–10.93]
Urinary and microbiological parameters	
Urinary nitrites positive	119 (49.6%)
Proteinuria	147 (61.3%)
Positive upper urinary tract culture	86 (35.8%)
Clinical outcomes	
ICU admission	30 (12.5%)
ICU length of stay, days	1.00 [0.00–3.00]
Vasopressor support	54 (22.5%)
In-hospital mortality	11 (4.6%)
Total hospital length of stay, days	7.00 [5.00–9.00]

Data are presented as mean ± standard deviation, median [25th–75th percentile], or *n* (%), as appropriate. NEWS2 = National Early Warning Score 2; eGFR = estimated glomerular filtration rate; ICU = intensive care unit.

**Table 3:** Comparison of admission characteristics, inflammatory markers, and clinical outcomes between culture-positive and culture-negative patients.

Variable	Culture-Negative (*n* = 154)	Culture-Positive (*n* = 86)	*p*-Value
Demographic characteristics			
Age, years	55.71 ± 18.81	52.58 ± 20.18	0.230
Male sex	89 (57.8%)	45 (52.3%)	0.413
Body mass index, kg/m^2^	29.28 ± 6.32	28.87 ± 6.48	0.634
Rural residence	78 (50.6%)	47 (54.7%)	0.552
Medical history and comorbidities			
Previous kidney stones	75 (48.7%)	51 (59.3%)	0.115
Diabetes mellitus	78 (50.6%)	50 (58.1%)	0.265
Hypertension	69 (44.8%)	47 (54.7%)	0.143
Chronic kidney disease	30 (19.5%)	13 (15.1%)	0.398
Previous urinary tract infection	12 (7.8%)	4 (4.7%)	0.350
Hospitalization within the last 30 days	21 (13.6%)	8 (9.3%)	0.323
Admission characteristics			
NEWS2 score	4.00 [3.00–5.00]	5.00 [3.00–6.00]	<0.001
Serum creatinine, mg/dL	1.14 [0.90–1.42]	1.21 [0.95–1.80]	0.164
Urea, mg/dL	39.40 [32.80–45.95]	43.55 [33.78–61.43]	0.062
eGFR, mL/min/1.73 m^2^	80.50 [58.00–103.25]	77.00 [47.25–102.00]	0.218
White blood cell count, /mm^3^	15,073.00 [12,875.00–20,208.75]	17,451.00 [14,256.25–21,760.00]	0.009
C-reactive protein, mg/L	102.50 [53.50–195.50]	149.50 [84.00–217.50]	0.028
Procalcitonin, ng/mL	2.09 [0.23–3.33]	11.08 [3.33–20.78]	<0.001
Key admission laboratory parameters			
Urinary nitrites positive	63 (40.9%)	56 (65.1%)	<0.001
Proteinuria	84 (54.5%)	63 (73.3%)	0.004
ICU admission	17 (11.0%)	13 (15.1%)	0.360
Vasopressor support	25 (16.2%)	29 (33.7%)	0.002
Total hospital length of stay, days	7.00 [5.00–9.00]	8.00 [5.75–10.00]	0.013

Values are presented as mean ± SD, median [IQR], or *n* (%). Continuous variables were compared using the independent-samples t-test or Mann–Whitney U test, and categorical variables using the χ^2^ test or Fisher’s exact test, as appropriate. NEWS2 = National Early Warning Score 2; eGFR = estimated glomerular filtration rate; ICU = intensive care unit.

**Table 4:** Univariable logistic regression analysis for predictors of positive upper urinary tract culture.

Predictor	Univariable OR	95% CI	*p*-Value
Age, years	0.992	0.978–1.005	0.230
Male sex	1.248	0.734–2.120	0.414
BMI, kg/m^2^	0.990	0.949–1.032	0.632
Rural residence	1.174	0.692–1.993	0.552
Previous kidney stones	1.535	0.900–2.618	0.116
Hypertension	1.485	0.874–2.523	0.144
Diabetes mellitus	1.353	0.795–2.304	0.265
Chronic kidney disease	1.359	0.666–2.769	0.399
Previous urinary tract infection	0.577	0.180–1.848	0.355
Serum creatinine, mg/dL	1.344	0.958–1.885	0.087
Urea, mg/dL	1.009	0.999–1.019	0.086
eGFR, mL/min/1.73 m^2^	0.994	0.985–1.002	0.160
White blood cell count, per 1000/mm^3^	1.057	1.004–1.112	0.033
C-reactive protein, mg/L	1.003	1.000–1.006	0.047
Procalcitonin, ng/mL	1.037	1.018–1.056	<0.001
NEWS2 score	1.183	1.061–1.320	0.003
Urinary nitrites positive	2.696	1.559–4.662	<0.001
Proteinuria	2.283	1.287–4.049	0.005

Univariable logistic regression was performed using positive upper urinary tract culture as the dependent variable. Results are reported as ORs with 95% CIs. For binary predictors, ORs represent the odds of culture positivity in patients with the characteristic present compared with those without it. For continuous predictors, ORs represent the change in odds per one-unit increase, except for white blood cell count, which is reported per 1000/mm^3^ increase. A *p*-value < 0.05 was considered statistically significant. NEWS2 = National Early Warning Score 2; eGFR = estimated glomerular filtration rate; BMI = body mass index; WBC = white blood cell count.

**Table 5:** Multivariable logistic regression model for positive upper urinary tract culture.

Predictor	Adjusted OR	95% CI	*p*-Value
Log_2_-transformed PCT (per doubling)	1.820	1.502–2.205	<0.001
NEWS2 score	1.167	1.014–1.344	0.031
Urinary nitrites positive	1.786	0.919–3.473	0.087
Proteinuria	1.076	0.535–2.166	0.837
WBC, per 1000/mm^3^	1.020	0.955–1.089	0.554
CRP, per 10 mg/L	1.006	0.966–1.047	0.784

Multivariable logistic regression was performed with a positive upper urinary tract culture as the dependent variable. Results are reported as ORs with 95% CIs. For continuous variables, ORs represent the change in odds per one-unit increase, except for white blood cell count (per 1000/mm^3^ increase) and C-reactive protein (per 10 mg/L increase). For binary variables, ORs represent the odds of culture positivity in patients with the characteristic present compared with those without it. NEWS2 = National Early Warning Score 2; WBC = white blood cell count; CRP = C-reactive protein.

**Table 6:** Exploratory cut-off-based analysis for positive upper urinary tract culture.

Predictor	Adjusted OR	95% CI	*p*-Value
PCT ≥ 3.0 ng/mL	10.881	5.215–22.702	<0.001
NEWS2 ≥ 5	2.802	1.419–5.536	0.003
Urinary nitrites positive	2.055	1.060–3.986	0.033
Proteinuria	1.348	0.665–2.733	0.408
WBC, per 1000/mm^3^	1.044	0.978–1.114	0.194
CRP, per 10 mg/L	1.011	0.971–1.054	0.591

PCT was categorized using the exploratory cut-off of ≥3.0 ng/mL identified by ROC analysis and the Youden index, while NEWS2 was categorized using the clinically relevant threshold of ≥5. Results are reported as adjusted odds ratios (ORs) with 95% confidence intervals (CIs). This secondary analysis was exploratory. PCT = procalcitonin; NEWS2 = National Early Warning Score 2.

**Table 7:** Incremental performance of models for positive upper urinary tract culture.

Model	Variables Included	AUC (95% CI)	Optimism-Corrected AUC	AIC
Model performance				
Model 1	WBC + CRP + urinary nitrites + proteinuria	0.691 (0.624–0.758)	0.672	297.0
Model 2	Model 1 + log_2_(PCT)	0.831 (0.780–0.883)	0.817	241.7
Model 3	Model 1 + NEWS2	0.720 (0.655–0.784)	0.696	293.4
Model 4	Model 1 + log_2_(PCT) + NEWS2	0.839 (0.788–0.889)	0.821	238.9
Incremental model comparisons				
Reference model	Expanded model	Added variable(s)	ΔAUC	DeLong *p*-value
Model 1	Model 2	log_2_(PCT)	0.140	<0.001
Model 1	Model 3	NEWS2	0.029	0.085
Model 2	Model 4	NEWS2	0.007	0.448
Model 3	Model 4	log_2_(PCT)	0.119	<0.001
Model 1	Model 4	log_2_(PCT) + NEWS2	0.148	<0.001

## Data Availability

The datasets generated and/or analyzed during the current study are available from the corresponding author upon reasonable request, subject to institutional approval and applicable patient confidentiality requirements.

## Abbreviations

The following abbreviations are used in this manuscript:

AUC	Area under the receiver operating characteristic curve
BMI	Body mass index
CFUs	Colony-forming units
CI	Confidence interval
CKD	Chronic kidney disease
CRP	C-reactive protein
eGFR	Estimated glomerular filtration rate
ICU	Intensive care unit
IQR	Interquartile range
NEWS2	National Early Warning Score 2
OR	Odds ratio
PCT	Procalcitonin
ROC	Receiver operating characteristic
SD	Standard deviation
SIRS	Systemic inflammatory response syndrome
UTI	Urinary tract infection
WBC	White blood cell count
